# Correction: Endobronchial valve positioning for alveolar-pleural fistula following ICU management complicating COVID-19 pneumonia

**DOI:** 10.1186/s12890-022-01845-y

**Published:** 2022-02-02

**Authors:** Pierluigi Donatelli, Fabiana Trentacosti, Maria Rosaria Pellegrino, Roberto Tonelli, Giulia Bruzzi, Alessandro Andreani, Gaia Francesca Cappiello, Dario Andrisani, Filippo Gozzi, Cristina Mussini, Stefano Busani, Gilda Valentina Cavaliere, Massimo Girardis, Elisabetta Bertellini, Enrico Clini, Alessandro Marchioni

**Affiliations:** 1 University Hospital of Modena, Respiratory Diseases Unit, Department of Medical and Surgical Sciences, University of Modena Reggio Emilia, Modena, Italy; 2 Clinical and Experimental Medicine PhD Program, University of Modena Reggio Emilia, Via Università 4, 41121 Modena, Italy; 3 University Hospital of Modena, Infectious Diseases Unit, University of Modena Reggio Emilia, Modena, Italy; 4 University Hospital of Modena, Anesthesiology Unit, University of Modena Reggio Emilia, Modena, Italy; 5 Laboratory of Experimental Pneumology, Modena, Italy; 6 Respiratory Diseases Unit and Center for Rare Lung Disease, Department of Surgical and Medical Sciences, University Hospital of Modena, Via del Pozzo, 71, 41125 Modena, Italy

## Correction to: BMC Pulm Med (2021) 21:1-6 10.1186/s12890-021-01653-w

Following publication of the original article [1], the authors flagged that the author name Fabiana Trentacosti had been erroneously spelled as Fabiana Trenatacosti.

The published article has since been corrected.

The authors apologize for any inconvenience caused.

